# Cutaneous Plasmablastic Lymphoma in an Immunocompetent Patient with Long-Term Pyrimethamine Use for Essential Thrombocythemia: A Case Report and Literature Review

**DOI:** 10.1155/2013/541783

**Published:** 2013-02-06

**Authors:** Ing Soo Tiong, Magreet Strauss, Michael B. Y. Lau, Shingirai Chiruka

**Affiliations:** ^1^Southern Blood and Cancer Service, Dunedin Hospital, Private Bag 1921, Dunedin 9054, New Zealand; ^2^Division of Haematology, Southern Community Laboratories, Dunedin 9016, New Zealand

## Abstract

We report a case of Epstein-Barr-virus-(EBV-) positive primary cutaneous plasmablastic lymphoma in a human-immunodeficiency-virus-(HIV-) negative, immunocompetent 62-year-old female patient. We postulate that her lymphoma development is due to the longstanding use of pyrimethamine for essential thrombocythemia. This has never been described in the literature.

## 1. Case Report

A 62-year-old woman was initially diagnosed with essential thrombocythemia (ET) on the basis of bone marrow aspirate and trephine at age 36 (November 1986), which was later proven to be Janus kinase 2 V617F mutation (JAK2) positive. She was started on hydroxyurea in July 1987 due to platelet counts of >1000 × 10^9^/L and recurrent transient neurological symptoms. This was subsequently changed to pyrimethamine in September 1994 due to concerns about the leukemogenic risk of hydroxyurea. She otherwise maintained good health other than a history of osteoporotic T8 compression fracture. 

**Figure 1:**
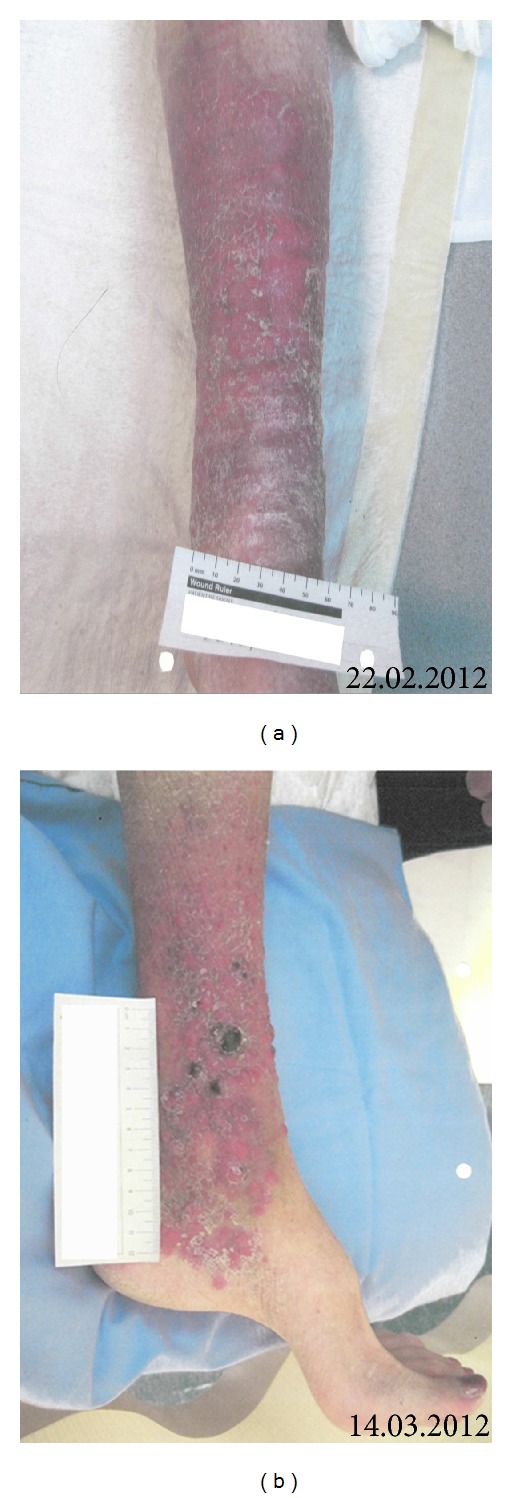
Skin.

In February 2012, she presented to our service with a 3-month history of fevers, weight loss, and lethargy. She also noted a general discomfort and brownish discoloration in her left leg during the same period (Figure 1(a)), which was managed by the vascular surgeons as venous insufficiency. Her peripheral blood film showed leukoerythroblastic changes. Bone marrow aspirate and trephine biopsy showed features consistent with ET including megakaryocytic hyperplasia and megakaryocytes of large complex nuclei. There was no evidence of myelofibrotic or acute leukemic transformation. 

Shortly after the previously mentioned, she was admitted under the respiratory team with an atypical pneumonia. During the inpatient stay, she noticed increasing nodular appearance on her leg. On physical examination, she had multiple nodular lesions extending from mid shin to ankle (Figure 1(b)). There was no ulceration, bleeding, or discharge from the lesions. There was no palpable lymphadenopathy or hepatosplenomegaly. A punch biopsy of the lesion was subsequently performed. 

**Figure 2:**
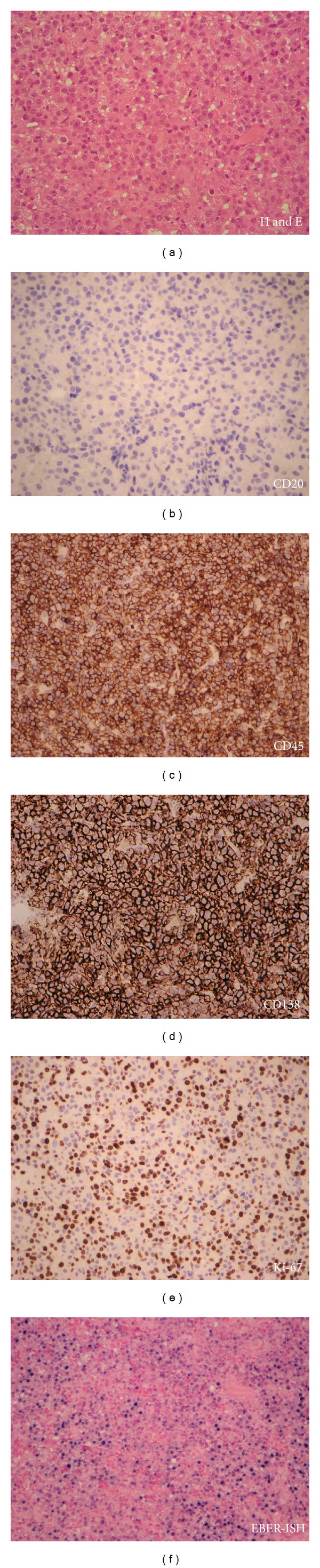
Punch biopsy of the left leg.

The biopsy showed a diffuse dermal infiltrate of large pleomorphic cells, some with plasmacytic differentiation (cells with rounded nuclei, coarser chromatin, and smaller nucleoli) and others with the appearance of immunoblasts (cells with enlarged nuclei, vesicular chromatin, and a single prominent nucleolus) (Figure 2(a)). Immunohistochemistry showed these to be negative for CD20 and stain positive for CD138, Bcl-2, CD45, and CD79a (weak), with a high Ki-67 proliferation index approaching 50% (Figures 2(b) to 2(e)). Pan-cytokeratins and melanoma markers (S-100, Melan-A) were negative, as was the staining for other lymphoid markers (CD2, CD56, CD30, Bcl-6, and TdT). Staining for Epstein-Barr virus (EBV) by in situ hybridization (EBV EBER-ISH) showed positive nuclear staining (Figure 2(f)). This was consistent with the diagnosis of plasmablastic lymphoma.

**Figure 3:**
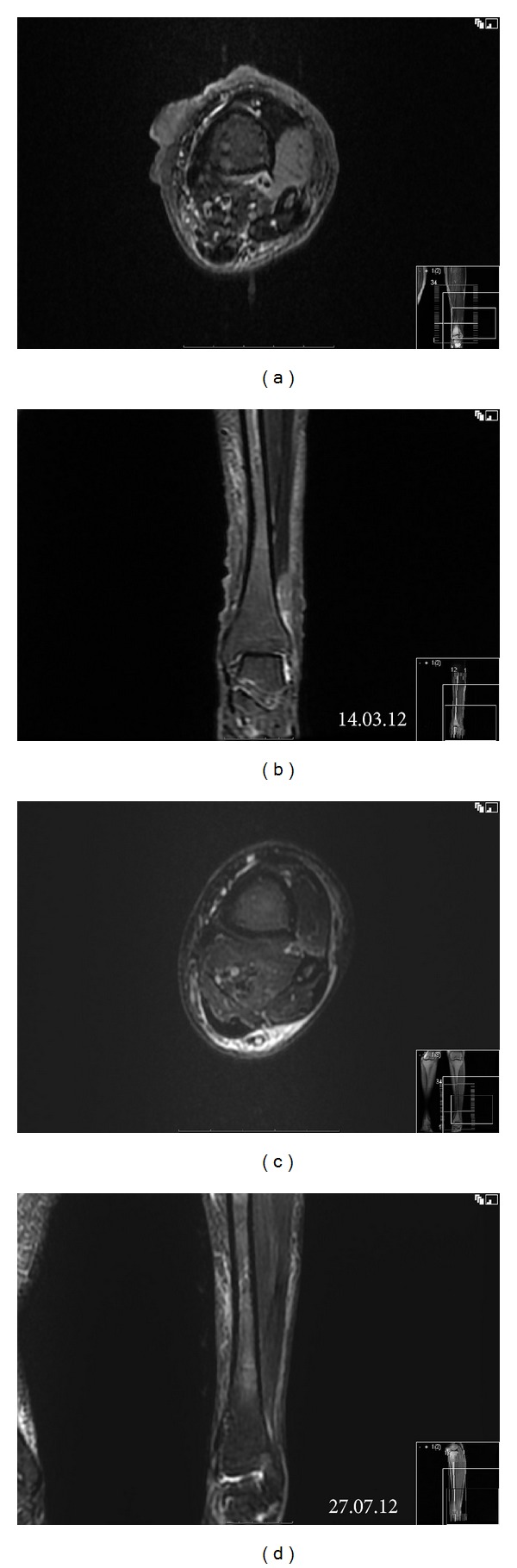
MRI of the left leg. Subcutaneous nodules and myositis of tibialis anterior are shown on T2 fat-saturated axial view (a) and short T1 inversion recovery (STIR) coronal view (b). After treatment, the changes are resolved (c, d).

**Figure 4:**
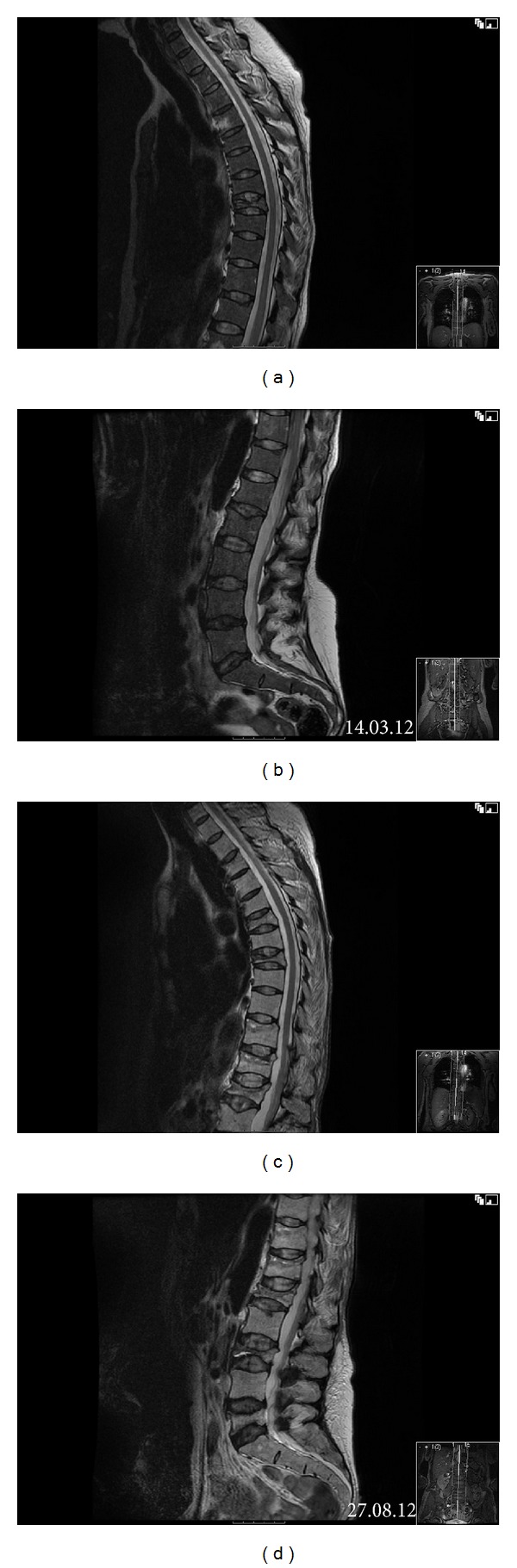
MRI spine. T2 fat-saturated axial view at the initial staging showed a compression fracture of T8 (a) and no other osseous lesions (a, b). Five months later, appearances were suspicious for lymphomatous infiltration with abnormal signal in T11 and T12 vertebral bodies and multilevel compression fractures of thoracic spine (c) and lumbar spine (d).

Staging investigation including a magnetic resonance imaging (MRI) of the left leg confirmed the subcutaneous lesions and adjacent myositis of tibialis anterior without any bony involvement (Figures 3(a) and 3(b)). MRI of the spine showed the T8 compression fracture with no other suspicious osseous lesions (Figures 4(a) and 4(b)). Whole body computed tomography (CT) scan revealed no other site of disease other than a mild splenomegaly at 16.2 cm. 

Other relevant investigations included hemoglobin 106 g/L (115–155 g/L), platelet 123 × 10^9^/L (150–430 × 10^9^/L), neutrophils 8.7 × 10^9^/L (1.9–7.5 × 10^9^/L), LDH 1151 IU/L (85–225 units/L), *β*_2_-microglobulin 6.51 mg/L (1.00–3.50 mg/L), IgA kappa monoclonal protein 7 g/L, and IgG kappa monoclonal protein <1 g/L. Patient was serologically negative for hepatitis B, hepatitis C, and human immunodeficiency (HIV) viruses. There was serological evidence of past EBV and cytomegalovirus exposure. 

This confirmed a diagnosis of stage 1B primary cutaneous plasmablastic lymphoma in a HIV-negative host without evidence or history of immunosuppression. The pyrimethamine was stopped, and combination chemotherapy with cyclophosphamide, doxorubicin, vincristine, and prednisone (CHOP) was started every three weeks. After the first cycle of the CHOP her platelet count started rising as the pyrimethamine had been stopped. Rather than introducing another antiproliferative agent, she was changed to CHOP every 14 days with pegylated growth factor support. This effectively controlled the platelet counts. She completed 6 cycles of chemotherapy which was then followed by a course of consolidation radiotherapy with 3600 cGy over 18 fractions. This resulted in a near resolution of the skin nodules and the myositis on the repeat of MRI scan (Figures 3(c) and 3(d)). 

However, patient developed increasing back pain in August 2012. MRI spine showed multilevel compression fractures at T6, T8, L1, L3, and L5 as well as abnormal marrow signal at T11 and T12 suspicious for lymphomatous infiltration (Figures 4(c) and 4(d)). Rather than having vertebral biopsies, due to frailty, she was treated with a single fraction (600 cGy) of radiotherapy. A CT scan of chest, abdomen, and pelvis at this time did not demonstrate any lymphadenopathy.

At the time of writing, she is still alive, 10 months after the diagnosis of PBL, and has complete resolution of the leg lesion. Her back pain is controlled with moderate amount of opiates. Platelet counts remain controlled without an antiproliferative agent. 

## 2. Discussion

Plasmablastic lymphoma (PBL) is a relatively new clinical entity described as a separate entity from diffuse large B-cell lymphoma [1]. It was initially described in HIV-infected individuals, involving the oral cavity [2]. However, cases of PBL involving extraoral sites are becoming increasingly recognized, particularly in HIV-negative patients [3–5]. 

Immunophenotyping is an important adjunct in differentiating PBL from other neoplasms. PBL expresses a plasma cell phenotype with positive CD138, CD38, Vs38c, and IRF4/MUM1 and are negative or only weakly positive for CD45 and CD20 (and PAX5). CD79a is positive in approximately 50–85% of the cases. Ki67 index is usually very high. EBV EBER is positive in up to 75% of the cases [1].

Important morphological differentials in this case would include diffuse large B-cell lymphoma (DLBCL), not otherwise specified; primary cutaneous diffuse large B-cell lymphoma, leg type; plasma cell myeloma or plasmacytoma; myeloid sarcoma; and metastatic melanoma. In this case, both forms of DLBCL are excluded due to the negative CD20. Plasma cell neoplasms are typically monomorphic in appearance and usually express CD56 and lack of EBV expression. MPO and S-100 negativity rule out myeloid sarcoma and metastatic melanoma, respectively.

To date, 25 cases of PBL presenting in the skin have been reported [6–31], with male predominance (*n* = 17 or 68%), and over half were HIV positive (*n* = 14 or 56%). Excluding the HIV-positive hosts, the gender distribution becomes much move even, with 6 males and 6 females. Interestingly, half of the cases had presentation involving the lower limb (12/24 or 50%). EBV was implicated in 80% (16/20) of the cases. At diagnosis, 10 cases had localized cutaneous PBL without systemic disease, whereas 8 cases had systemic involvement at diagnosis. No information could be ascertained about systemic involvement in 7 other patients. 

Most of the previously mentioned patients were immunosuppressed with HIV infection (*n* = 14), posttransplantation (*n* = 8), or autoimmune disorder (*n* = 1). Two patients, however, were immunocompetent hosts without any history of acquired or iatrogenic immunosuppression. These two patients were an 80-year-old male [7] and an 86-year-old female [26], respectively. To our knowledge, our patient would represent the third reported case of cutaneous PBL in an immunocompetent host. 

Liu et al. [32] recently reported a series of 10 patients with PBL who were negative for HIV and had no other known immunodeficiency conditions, other than advanced age (median age = 68 years). Of note, none of these cases presented with skin lesions. The term plasmablastic lymphoma of the elderly (PBL-E) has been coined to account for the immunosenescence state found in these patients. This bears several similarities to age-related EBV-associated B-cell lymphoproliferative disorder (AR-EBV LPD), which was newly listed in the 2008 World Health Organization classification of lymphoid neoplasms. 

One unique feature of this case is the long-standing use of pyrimethamine, a dihydrofolate reductase inhibitor. It is rarely used for myeloproliferative disorder in this era due to other more effective treatments. Its main use currently is for prevention and treatment of malaria and toxoplasmosis. We suspect that it might be involved in the immune dysregulation and thus the pathogenesis of her PBL. Animal studies demonstrated that pyrimethamine could enhance antibody responses to sheep red blood cell, augment delayed-type hypersensitivity response [33, 34], inhibit signal transducer and activator of transcription 3 (STAT3) pathway [35], and induce apoptosis of several cell lines [36–38]. Little is known about the effect of long-term pyrimethamine use on immune status. We could only identify a single case of non-Hodgkin's lymphoma developing in the gut in a patient while being on pyrimethamine [39]. It can rarely cause a pseudolymphoma drug reaction [40]. We could not identify any reported cases of PBL in association with myeloproliferative disorder. 

Her previous use of hydroxyurea is unlikely to be associated with the current presentation. It was discontinued 18 years ago. The concerns regarding leukemogenicity were largely disproved in larger studies about the use of hydroxyurea in ET [41–43] and sickle cell disease [44]. We could not identify any reported cases of PBL in association with hydroxyurea. 

In conclusion, we present a case of primary cutaneous PBL in the setting of longstanding pyrimethamine use for a myeloproliferative disorder. It is quite possible that the long-term pyrimethamine use resulted in a dysregulated immune system and the occurrence of PBL in this otherwise healthy 62-year-old female. Age-related immunosenescence may have played a role.
